# A critical analysis of the decreasing trends in tuberculosis cure indicators in Brazil

**DOI:** 10.36416/1806-3756/e20240186

**Published:** 2024-08-07

**Authors:** Hinpetch Daungsupawong, Viroj Wiwanitki

**Affiliations:** 1. Private Academic Consultant, Phonhong, Lao People’s Democratic Republic.; 2. University Centre for Research & Development Department of Pharmaceutical Sciences, Chandigarh University, Mohali, Punjab, India.

## DEAR EDITOR:

“A critical analysis of the decreasing trends in tuberculosis cure indicators in Brazil, 2001-2022”^1^ is an interesting article. In brief, the study examined the longitudinal development of tuberculosis cure indicators between 2001 and 2022 in Brazil. The data show that, throughout most of the country, there was a considerable decline in the cure indicators for patients with pulmonary tuberculosis or tuberculosis/HIV coinfection, as well as for those undergoing tuberculosis retreatment. In addition, according to national statistics, the mean annual percentage change in cure rates for those categories showed a consistent downward trend. These results point to a worrisome pattern that calls into question the efficacy of the current tuberculosis treatment programs in the country.

The study has some potential flaws, because it relies too heavily on administrative data, which may not be entirely accurate or complete. To ensure the validity of the findings, the study could have profited from adding other data sources or validation techniques. In addition, individual-level characteristics that may affect the effectiveness of tuberculosis treatment may not be fully taken into account by an ecological time-series design. In order to gain a deeper understanding of the factors influencing the observed trends in cure indicators, future studies could incorporate more thorough analyses at the individual level.

Further investigation into the effects that socioeconomic variables, health care service accessibility, and the caliber of tuberculosis treatment programs have on cure rates in Brazil could be an avenue for future research. The efficacy of the efforts to control tuberculosis in the country could be increased by identifying the obstacles to effective treatment and devising solutions to overcome them. Research endeavors could also concentrate on assessing the execution of particular measures or regulations aimed at improving tuberculosis treatment results, such as closely monitored therapy and patient assistance initiatives. Future studies may be able to fill these gaps and help create more focused and efficient methods of controlling tuberculosis in Brazil.
