# Supplementary material for: A critical analysis of the decreasing trends in tuberculosis cure indicators in Brazil
Source: J Bras Pneumol. 2024 Aug 7;50(4):e20240186. doi: 10.36416/1806-3756/e20240186 (PMC11449622; doi:10.36416/1806-3756/e20240186)
Supplement: Related texts

# Authors’ reply

**Authors:** Gabriel Pavinati, Lucas Vinícius de Lima, Pedro Henrique Paiva Bernardo, Jhenicy Rubira Dias, Bárbara Reis-Santos, Gabriela Tavares Magnabosco

**Affiliations:** 1. Programa de Pós-Graduação em Enfermagem, Universidade Estadual de Maringá, Maringá (PR) Brasil.; 2. Programa de Residência em Enfermagem, Universidade Estadual de Londrina, Londrina (PR) Brasil.; 3. Rede Brasileira de Pesquisa em Tuberculose - Rede TB - Rio de Janeiro (RJ) Brasil.

We appreciate the attention our recently published article in the *Jornal Brasileiro de Pneumologia* ^1^ has received. Our goal in addressing the concerning trend toward a decease in tuberculosis cure indicators in Brazil was to stimulate debate on this subject. However, appropriate conceptual definitions are crucial to ensure that the debate is appropriate. Albeit a suitable design to respond to the objective of the study, an ecological approach has some limitations. It is crucial to avoid the fallacy of attributing collective inferences to the individual level, and it was beyond the scope of the study to infer effectiveness or efficacy of tuberculosis control programs. We hope our study stimulates further detailed research and discussions.

We concur that the body of knowledge on the subject is still evolving. We recognize that our findings present a valuable opportunity to guide new, original, and unprecedented research. This approach can be pursued from various angles, such as using an ecological design that incorporates contextual and programmatic variables to evaluate their influence on the primary outcome and employing individual data sources (e.g., cohort and case-control studies) to develop a framework underpinning all tuberculosis control actions in the country. Continued research from diverse perspectives is essential for a comprehensive understanding of tuberculosis.

Concerns surrounding the utilization of administrative data in our research, particularly those relating to underreporting and filling errors within the information system, were acknowledged as factors that could influence the interpretation of the results^1^. However, this information system has been extensively studied, and the consistency of its attributes has been confirmed in previous studies^2^. Consequently, we are confident that this did not affect the results presented, which are also supported by operational publications^3^. Addressing data accuracy and reliability is vital for robust conclusions in tuberculosis research and policy development.

Discussions like these underscore the importance of addressing the complex problem of tuberculosis in depth and diligently. We believe that suggestions to explore other data sources and consider individual and contextual factors-as we partially did by segmenting by occurrence location and specific groups (e.g., people with pulmonary tuberculosis, people living with HIV, and people in retreatment)-are pertinent. They can unveil unexplored knowledge and contribute to eliminating the epidemic in our country. Ongoing dialogue and research are crucial for effective tuberculosis control strategies and achieving public health goals in Brazil.

Finally, it is worth reiterating that our findings indicated a concerning trend in the Brazilian context^1^ ^,^ ^3^: a decline in tuberculosis cure indicators. Therefore, we hope our article will continue to spark debate on the topic and serve as a launching pad for more detailed studies and the development of more assertive and robust policies. By acknowledging the existing reality, we can point out paths, trajectories, and trends. We should also consider the efforts of the Brazilian National Tuberculosis Program to implement policies and actions that bring us closer to the desired goal of eliminating tuberculosis as a public health problem-a commitment shared by all.

## References

[1] Pavinati G, Lima LV, Bernardo PHP, Dias JR, Reis-Santos B, Magnabosco GT (2024). A critical analysis of the decreasing trends in tuberculosis cure indicators in Brazil, 2001-2022. J Bras Pneumol.

[2] Silva GDMD, Bartholomay P, Cruz OG, Garcia LP (2017). Evaluation of data quality, timeliness and acceptability of the tuberculosis surveillance system in Brazil's micro-regions Cien Saude. Colet.

[3] Brasil (2023). Ministério da Saúde. Secretaria de Vigilância em Saúde e Ambiente. Departamento de HIV/Aids, Tuberculose, Hepatites Virais e Infecções Sexualmente Transmissíveis. Coordenação-Geral de Vigilância da Tuberculose, Micoses Endêmicas e Micobactérias Não Tuberculosas. Boletim Epidemiológico - Tuberculose 2023.
